# Structure-based analysis of curcumin and conventionaldrugs targeting tumor-inducing protein PHF20

**DOI:** 10.6026/97320630014477

**Published:** 2018-11-03

**Authors:** Vibha Agrawal, Aradhya Mishra, Shivani Tiwari, Kumar Srivastava Akhileshwar

**Affiliations:** 1 Department of Biotechnology, Sri Agrasen Kanya P.G. College, Bulanala, Varanasi - 221003, India

**Keywords:** Curcumin, conventional drugs, PHF20, molecular docking

## Abstract

Recently, the PHF20 has been reported as tumor inducer protein by suppressing the activity of tumor suppressor protein p53. Conventional drugs (albendazole, doxazosin, and propranolol) are used for treatment of cancer causing side effect. The secondary metabolite curcumin is employed in various diseases treatment including cancer. The present study is to explore curcumin in comparison to selected conventional drugs by using molecular docking. The online database “Molinspiration online server” detected the physicochemical pharmacokinetics and drug likeness score of curcumin and conventional drugs. Results from Molinspiration online server showed that curcumin did not violate the “Lipinski five rule” for drug. The lead compound for molecular docking exhibited the potential interaction to the active site of PHF20. The resulted binding energy of albendazole and doxazosin were -21.97 and -26.64 respectively. The binding energy (-18.12 kcal/mol) of curcumin was higher than propranolol (17.62 kcal/mol). Thus, curumin has greater potential to interact for further consideration as an anti-cancerous regimen

## Background

PHF20 protein is associated with lysine acetyltransferase complex responsible for acetylation of histone H4 and non-histone proteins that is participated in transcriptional regulation and Ataxia telangiectasia mutated (ATM)-dependent DNA damage response 1-2. The complex is made of a WD repeat domain 5 (WDR5) subunit with H3K4-specific methyl-transferase and MLL1 complex 2-3. The activities of lysine acetyltransferase complex is essential for activation of transcriptional genes, which evident is reached out after a synergistic distribution of H3K4me and H4K16ac marks at promoters regions 4. The genomic and biochemical studies explain that lysine acetyltransferase complex induces to MLL1 activity, promoting dimethylation of H3K4 in an acetylationdependent manner 4. PHF20 has been identified as an antigen in glioblastoma patients that is highly expressed in several types of cancers and imparted in the development and progression of glioma, adenocarcinomas, and lung cancer 5. NF-kB is activated in some tumor due to highly expression of PHF20 6. PHF20 knockout mice have very short life span and exhibits different types of phenotypes within the skeletal and hematopoietic systems 7. PHF20 deficient somatic cells are not able to convert into induced pluripotent stem cells (iPSCs) and showing a requirement of this factor for cell reprogramming 3. The molecular mechanism of PHF20 attributes to transcription and p53 regulation for survival and carcinogenic activity.

Albendazole suppresses the proliferation of cancer cells including a hepatocellular cancer cells, colorectal cells, ovarian cells, pancreatic cells, and other malignant human cell lines. It has been reported in some patients with colorectal cancer and liver metastases exhibited a reduction of tumor markers (CEA) after oral administration of albendazole 8. Doxazosin stimulates apoptosis by arresting cell cycle of GB cells and suppresses the hERG protein expression through siRNA-mediated knock down mimicked pro-apoptotic effects of doxazosin. The HERG is binding receptor for doxazosin, does not affect on cell viability attenuated doxazosin-induced apoptosis of GB cells 9. The propranolol induces apoptosis in PC-2 cells and stimulates proteolytic activities of caspase 3 and caspase 9, whereas procaspase 8 have no cleavage fragments indicating that apoptosis in PC-2 cells is induced through intrinsic apoptotic pathway 6.

Curcumin is a polyphenol compound isolated from the rhizomes of Curcuma longa L. that is frequently used as traditional medicine, cuisine and the food industry worldwide. Curcumin plays a vital role in several diseases like anti-inflammatory, antimicrobial, lipid modifying, anticancer, and antiangiogenic. Many studies reported that the curcumin acts as a chemopreventive agent against different types of human cancers like breast, liver, hematological, gastrointestinal, prostate, and brain cancers. Curcumin could modulate effectively to the expression of various genes involving in different stages of proliferation, invasion, angiogenesis, and metastasis of cancer cells. Curcumin suppresses to tumor progression and metastasis via blocking the various types of signal transduction pathways like p53, Ras, Wnt-β, MAPKs, ERK, PI3K, and Akt in metastatic cells 10. The present study based on molecular docking was undertaken to investigate the anticancerous potential of curcumin in comparison to conventional drugs (albendazole, doxazosin, and propranolol) targeting PHF20 protein inducing for cancerous cells.

## Methodology

### Selection of compounds and oncoprotein

**Figure 1:**
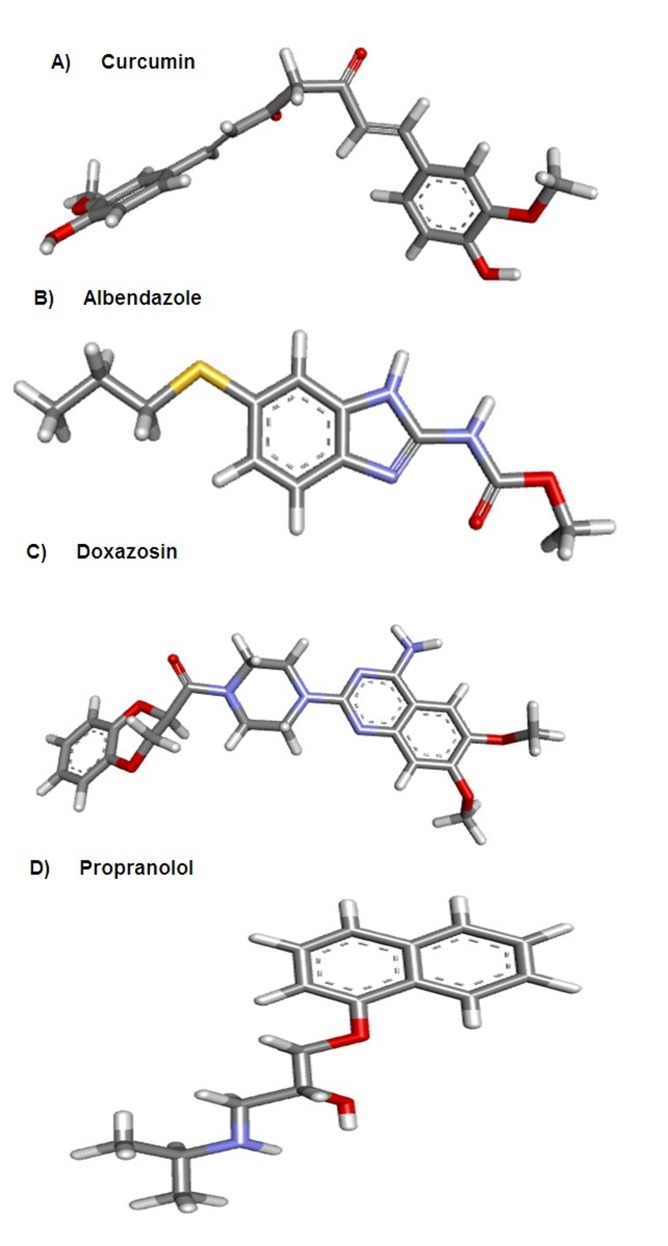
The 3D-structure of selected compounds

The 3D-crystal structure of curcumin PubChem CID: 969516 (Figure 1a) and conventional drugs: Albedazone (PubChem CID: 2082) (Figure 1b), Doxazosin (PubChem CID: 3157) (Figure 1c), Propranolol (PubChem CID: 4946) (Figure 1d) were retrieved from the molecular information repository PubChem search engine (PubChem) 11 for determination of their pharmaceutical potential.

**Figure 2:**
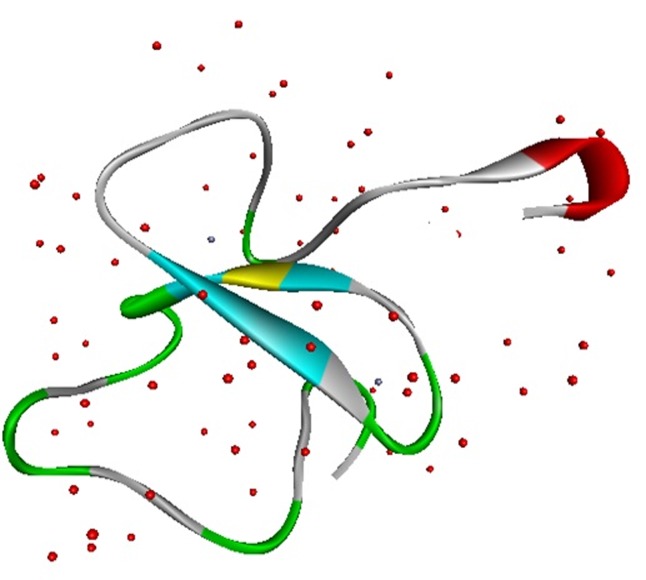
3D-structure of targeting tumor-inducing protein PHF20

The 3D-crystal structure of PHF20 with different confirmations has been explained in a literature 5. The protein structure file of PHF20 (PDB ID: 5TAB) (Figure 2) with resolution: 1.25 Å was downloaded from the RSCB Protein Data Bank (PDB). The PHF20 protein with amino acids length (53) has R-factor: 0.118 and R-free 0.143.

### Analysis of physicochemical pharmacokinetics of lead compounds

Molecular properties like membrane permeability and bioavailability of lead compounds depend on some basic properties of molecules like partition coefficient (logP), molecular weight (MW), and number of hydrogen bond acceptors/donors that is associated with Lipinski ‘‘rule of five’’. Lipinski’s rule elucidates that a molecule with good membrane permeability has MW 500 and hydrogen bond donors 5 and acceptors 10. In the current study, Molinspiration online server was employed to analyze the druglike properties of lead compounds 12.

### Molecular Docking of Compounds

Molecular docking experiment was implied to investigate the interactive mode of curcumin and conventional drugs (albedazone, doxazosin, and propranolol) to PHF20 protein. PatchDock web server was used for ligand-receptor docking. About 1000 solutions with score, area, and six-dimensional transformation space were obtained from Patch-Dock server, and then all solutions were subsequently transferred into FireDock to refine the ten best solutions associated with global energy. The obtained complexes from FireDock were ranked according to minimum global binding energy. Eventually, the Discovery Studio 4.0 Client was used for the visualization of binding modes of the receptor and ligands 13.

## Results

Table 1 shows all compounds followed the rule of five indicating the good bioavailability of molecules (curcumin, albedazol, doxazosin, and propanlol). The druglikeness score of lead molecules are determined with combination of GPCR, ion channel modulator, kinase inhibitor, nuclear receptor ligands, protease inhibitor, and enzyme inhibitor, which has been applied to investigate the efficiency of molecules to qualify for drug development. Srivastava et al. (2015) elucidated that the larger the bioactivity score has higher probability of the specific molecule to be active 13. If bioactivity score of molecule is greater than 0.00, has considerable biological activities and score between 0.50 to 0.00 are considered to be moderately active and if value is less than 0.50 it is presumed to be inactive 13. The obtained values of drug likeness score revealed that curcumin followed the good druglikeness score (>0.50) along with other standard drugs like albedazol, doxazosin, and propanlol (Table 2).

**Figure 3:**
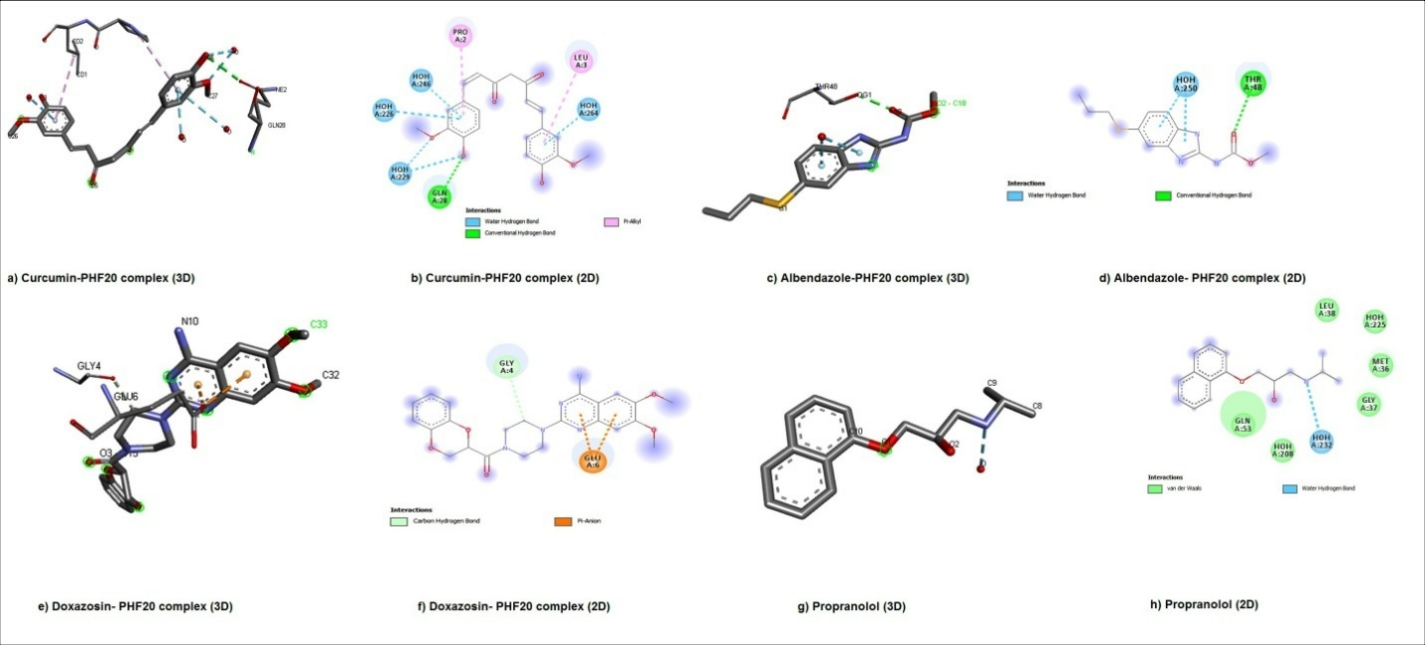
Visualization of 3D (a, c, e, and g) and 2D (b, d, f, and h) structures of the interacting residues of PHF20 protein with selected compounds

The lead compounds interacted to target protein PHF20 which mode of binding have been presented in 3D [Figure 3 (a, c, e, and g)] and 2D [Figure 3 (b, d, f, and h)] structures. Curcumin made complex to PHF20 by interacting with residues (GLN28, PRO2, and LEU3) (Figure 3). The residue (GLN28) of PHF20 made H-bond interaction to curcumin and other residues (PRO2 and LEU3) had Pi-alkyl interactions as shown in Figure 3. Figure 3 showed for 3D/2D crystal structure of complex that the albedazone attached to single residue THR48 of PHF20 through H-bond interactions. In complex of doxazosin-PHF20, the residues (GLU4 and GLU6) were involved in interaction to doxazosin (Figure 3). The 2D-structure of complex Doxazosin-PHF20 revealed the carbon-hydrogen bond interaction in GLY4 of PHF20 and Pi-Anion interaction was observed in GLY6 (Figure 3). The 3D and 2D propanlol complex exhibited the van der Waals interactions to residues (LEU38, MET36, GLY37, and GLN53) of PHF20 protein as shown in Figure 3 (g and h).

## Discussion

The ADME (absorption, distribution, metabolism, and elimination) property for molecules has been assigned for drug development process. The molecular properties and bioactivity of the lead compounds were obtained through online data server Molinspiration and had logP values along with other physiochemical properties (molecular mass, the number of hydrogen bond acceptors, and donors) (Table 1). The compounds which are violating any one of the Lipinski’s rule may create problems in bioavailability 14. The clinical trials of several compounds as drug have been failure due to lack of potential interactive properties against the desired drug target 15. It has been observed that the pharmacokinetics of molecules is directly blamed in more than half of clinical trials. Curcumin remains stable at acidic condition and has slow degradation at pH 1-6 and normally encountered in stomach 16. The pharmacokinetic properties of curcumin have been assessed to overcome the problems existing with interactive potential of conventional drugs against drug target (Table 2). The current study have examined the overall drug likeness score for curcumin along with conventional drugs (albendazole, doxazosin, and propranolol) that are being considered for PHF20 onco-protein target. 

Molecular docking of curcumin agonist ligand into the binding site of the active-state of PHF20 confirmation resulted in binding energy (-18.12 kcal/mol) that was higher than interactive residues (LEU38, MET36, GLY37, and GLN53) of propanlol (-17.62 kcal/mol) (Table 3), suggesting high binding affinity toward residues of (GLN28, PRO2, and LEU3) of PHF20. Srivastava et al. (2015) reported that the curcumin have potential to interact with oncongenic factors like CagA protein. Albedazone is bound to active residue (THR48) of PHF20 protein with binding energy (- 21.97 kcal/mol) (Table 3) 13 and structure-based study on albedazone are efficient tool for exploring the potentiality to disrupt the natural integrity of oncoprotein 17. The obtained binding energy (-26.64 kcal/mol) of doxazosin from molecular docking was highest among selected lead compounds with interactions of active residues (GLU6 and GLU4) of PHF20. Petty et al. (2018) suggested that the doxazosin has higher binding affinity toward the receptor, which could alter the native structure of oncogenic protein 18.

## Conclusion

The present work was designed to explore curcumin in comparison to conventional drugs (albedazol, doxazosin, and propanlol) against tumor inducing protein PHF20. Data show higher binding energy of curcumin than propanlol against tumor inducing protein PHF20 protein could be used as a conventional drug without producing side effect. Results show that molecular docking could be used as an efficient tool by which the potentiality of unexplored natural product could be indentified against severe diseases including cancer.

## Figures and Tables

**Table 1:** Analysis of Physicochemical pharmacokinetics of compounds by molinspiration online server

Details	Curcumin	Albedazole	Doxazosin	Propanlol
Octanol–water partition coefficient	2.303	2.57	2.08	2.97
Polar surface area	93.066	67.02	112.28	41.49
Number of nonhydrogen atoms	27	18	33	19
Molecular weight	265.34	368.385	451.48	259.35
Number of hydrogen-bond acceptors (O and N atoms)	6	5	10	3
Number of hydrogen-bond donors (O and N atoms)	2	2	2	2
Number of rule of five violations	0	0	0	0
Number of rotatable bonds	8	5	4	6
Molecular volume	234.09	332.182	395.9	257.82

**Table 2:** Determination of drug likeness score of compounds through molinspiration online server

Properties	Curcumin	Albedazone	Doxazosin	Propanlol
GPCR	-0.06	-0.11	0.13	0.12
Ion channel modulator	-0.2	-0.1	-0.3	0.06
Kinase inhibitor	-0.26	-0.04	0.28	-0.17
Nuclear receptor ligand	0.12	-0.62	-0.52	-0.2
Protease inhibitor	-0.14	-0.18	-0.12	-0.04
Enzyme inhibitor	0.08	-0.02	0.07	0.04

**Table 3:** Estimation of binding energies of compound with interactive residues of PHF20 protein

Name of compounds	PubChem ID	Interacting residues	Global binding energy (kcal per mol)
Curcumin	969516	GLN28, PRO2, LEU3	-18.12
Albedazone	2082	THR48	-21.97
Doxazosin	3157	GLU6, GLU4	-26.64
Propanlol	4946	LEU38, MET36, GLY37, GLN53	-17.62
